# Supplementary material for: The spatial and social correlates of neighborhood morphology: Evidence from building footprints in five U.S. metropolitan areas
Source: PLoS One. 2024 Apr 10;19(4):e0299713. doi: 10.1371/journal.pone.0299713 (PMC11006153; doi:10.1371/journal.pone.0299713)
Supplement: Attachment — Submitted filename: PLOSONE_RevisionMemo 1_24.docx [file pone.0299713.s002.docx]

Reviewer #2: The authors carefully and meticulously responded to my previous concerns, and made targeted revisions to the article. The completeness of the whole content in this manuscript has been further improved. However, I think that the current abstract and introduction sections need further revisions to enhance their readability and comprehensibility.

1. The abstract section should at least provide some key information, such as the significance of your research field, research gap (what problems have not been solved, which could be your motivation and draws out your research questions), and some main results and findings (to support your conclusion and implications). Without these information, it is difficult to understand your methods and procedures (what are their purposes?) as well as your conclusion and implications (where do they come from?). In addition, there are too many “we do” sentences. I think some of them can be replaced by the description of your main results and findings.

**RESPONSE: Thank you for these helpful recommendations. We have revised the abstract to reduce the methodological discussion and incorporate a discussion of the research gap/motivation as well as main results. We have also removed multiple instances of the use of “we.” The revised abstract is provided here as well as in the text:**

**“Recent advances in quantitative tools for examining urban morphology enable the development of morphometrics that can characterize the size, shape, and placement of buildings; the relationships between them; and their association with broader patterns of development. Although these methods have the potential to provide substantial insight into the ways in which neighborhood morphology shapes the socioeconomic and demographic characteristics of neighborhoods and communities, this question is largely unexplored. Using building footprints in five of the ten largest U.S. metropolitan areas (Atlanta, Boston, Chicago, Houston, and Los Angeles) and the open-source R package, foot, we examine how neighborhood morphology differs across U.S. metropolitan areas and across the urban-exurban landscape. Principal components analysis, unsupervised classification (K-means), and Ordinary Least Squares regression analysis are used to develop a morphological typology of neighborhoods and to examine its association with the spatial, socioeconomic, and demographic characteristics of census tracts. Our findings illustrate substantial variation in the morphology of neighborhoods, both across the five metropolitan areas as well as between central cities, suburbs, and the urban fringe within each metropolitan area. We identify five different types of neighborhoods indicative of different stages of development and distributed unevenly across the urban landscape: these include low-density neighborhoods on the urban fringe; mixed use and high-density residential areas in central cities; and uniform residential neighborhoods in suburban cities. Results from regression analysis illustrate that the prevalence of each of these forms is closely associated with variation in socioeconomic and demographic characteristics such as population density, the prevalence of multifamily housing, and income, race/ethnicity, homeownership, and commuting by car. We conclude by discussing the implications of our findings and suggesting avenues for future research on neighborhood morphology, including ways that it might provide insight into issues such as zoning and land use, housing policy, and residential segregation.”**

2. The introduction section needs further enhancement to be more informative, more logical and better organized. For example, the 1st paragraph in the background section are redundant and repetitive (as the 1st paragraph of the introduction has the similar gist). The proposal of three research questions is abrupt, lacking sufficient groundwork of research background, literature review, and research gaps (some of which I can only find later in the text). In addition, I think you should emphasize your research contributions more plainly, especially the progress you have made compared to Jochem and Tatem (2021)’s work and other previous studies.

- the 1st paragraph in the background section are redundant and repetitive
- proposal of three research questions is abrupt, lacking sufficient groundwork of research background, literature review, and research gaps (some of which I can only find later in the text)
- I think you should emphasize your research contributions more plainly, especially the progress you have made compared to Jochem and Tatem (2021)’s work and other previous studies

**RESPONSE: Thank you for these helpful recommendations. We agree that emphasizing the paper’s contribution and how it differs from previous studies is important. We also that additional foregrounding of the importance of neighborhoods in social research was important for clarifying the motivation of the research and the gap in the existing literature prior to articulating the research question. These following two paragraphs in the introduction now address both of these issues:**

**“New data, tools, and techniques mean researchers are not limited to small case studies which have been common in urban morphology studies. Recent research using building footprints has used morphological analysis to characterize patterns of development at the neighborhood level [7]. For example, Jochem and Tatem use publicly available spatial datasets of building footprints to define their constituent elements (size, shape, and placement of structures) in England, Scotland and Wales and to examine the extent to which typologies of neighborhoods derived from unsupervised classification using building footprint morphometrics align with census-defined classifications for rural and urban areas of various types [7].**

**We adapt and extend this analysis to the U.S. context to analyze the dimensions and distribution of development inscribed in the morphology of neighborhoods in five of the ten largest U.S metropolitan areas and to develop a typology of U.S. neighborhoods based on their morphological characteristics. In doing so, we combine the tools of urban morphology with the theoretical contributions from a vast literature in urban studies, sociology, and planning that has explored how neighborhoods are a key mechanism that structures ecological, political and social outcomes in metro regions . Distinct types of neighborhoods (e.g., suburban enclaves, urban cores, rural districts) vary markedly in the characteristics of their population and the opportunities they provide [12–14]. Little is known, however, about whether the morphological characteristics measured by building footprints align with these pre-existing conceptual understandings of neighborhoods and the characteristics of residents in them. We address this gap in this study by answering three primary research questions: Can neighborhood-level estimates of building morphology be used to create a useful typology of U.S. neighborhoods that maps onto conceptual understandings of urban form? How does neighborhood morphology vary across the country and across central cities, suburban areas, and the urban fringe? Do neighborhoods with distinct building morphologies differ in regard to key socio-demographic characteristics?”**

**We appreciate the feedback regarding the short paragraph at the start of the Background section. We have shortened that discussion to prevent duplication.**
